# Prognostic Value of Ubiquitination-Related Genes in Ovarian Cancer and Their Correlation With Tumor Immunity

**DOI:** 10.1155/humu/8369299

**Published:** 2025-07-15

**Authors:** Shu Zhao, Xiaojing Lin, Yuying Huang, Zhongmin Kang, Huali Luo, Qizhu Zhang, Qinshan Li, Mengxing Li

**Affiliations:** ^1^Department of Obstetrics and Gynecology, Affiliated Hospital of Guizhou Medical University, Guiyang, Guizhou, China; ^2^Department of Obstetrics and Gynecology, Institute of Precision Medicine of Guizhou Province, Affiliated Hospital of Guizhou Medical University, Guiyang, Guizhou, China; ^3^Department of Obstetrics, Guizhou Provincial People's Hospital, Guiyang, Guizhou, China; ^4^Department of Clinical Biochemistry, School of Medical Laboratory Science, Guizhou Medical University, Guiyang, Guizhou, China; ^5^Clinical Medical College, Guizhou Medical University, Guiyang, Guizhou, China; ^6^Department of Hematology, Affiliated Hospital of Guizhou Medical University, Guizhou Province Institute of Hematology, Guizhou Province Laboratory of Hematopoietic Stem Cell Transplantation Centre, Guiyang, Guizhou, China; ^7^Department of Pathophysiology, Guizhou Medical University, Guiyang, Guizhou, China

**Keywords:** immune infiltration, ovarian cancer, prognostic model, TCGA, ubiquitination

## Abstract

Numerous studies have emphasized the importance of the ubiquitin–proteasome system (UPS) in the malignant progression of ovarian cancer (OC). However, whether ubiquitination-related genes (UbRGs) can be used to predict the prognosis of OC remains to be revealed. Patients with OC were divided into two clusters based on the expression of UbRGs, and prognosis was compared between the two clusters. A prognostic model was established based on UbRGs, and its predictive efficiency was validated using Kaplan–Meier (K–M) curves, receiver operating characteristic (ROC) curves, and a nomogram. Immune infiltration and gene mutation analyses were used to examine the effects of UbRGs on the prognosis of OC. The prognostic model served as a valid and independent predictor of OC prognosis. Immune infiltration revealed that the unique immune microenvironment of OC was regulated by UbRGs. Gene mutation analysis indicates that UbRGs likely influence OC malignant behavior by modulating gene mutation patterns. In addition, Ube2j1 was found to play an important role in regulating the malignant progression of OC. Furthermore, the mechanism by which Ube2j1 modulates the OC phenotype and reshapes its immune microenvironment via the JAK2/STAT3/PD-L1 pathway was elucidated, providing novel insights into the potential for ubiquitination-based immunotherapy in OC. This study provides novel insights into precision immunotherapy based on UbRGs in OC. The UbRGs-based prognostic model may help to provide novel insights for the application of ubiquitination-based immunotherapy in OC.

## 1. Introduction

Ovarian cancer (OC) remains among the fatal gynecological cancers, standing as the fifth most lethal cancer-associated death among women [1]. Within the spectrum of gynecological malignancies, OC is characterized by a notably high mortality rate, which can be attributed to its propensity for recurrence and late-stage detection [2]. The absence of early symptoms and effective screening methods leads to approximately 75% of cases being diagnosed at advanced stages, with around 70% of patients ultimately experiencing tumor recurrence [3]. Ovarian tumors encompass several histopathological subtypes, encompassing epithelial, germ cell, sex cord-stromal, and metastatic tumors. Of these, high-grade serous ovarian cancer (HGSOC) constitutes the most prevalent and aggressive, contributing to 70%–80% of mortality linked to OC. In recent decades, survival rates for HGSOC have improved marginally, with cytoreductive surgery combined with platinum-based agents and paclitaxel remaining the standard first-line treatment. However, despite an initially favorable response, recurrence occurs in the majority of patients [4]. Consequently, the development of alternative therapeutic strategies remains a critical priority.

Post-translational modifications facilitate the rapid response and adaptation of organisms to environmental fluctuations. Ubiquitin, a predominant modifier of proteins post-translationally, attaches to lysine residues of various cellular proteins, thereby serving key regulatory functions in numerous essential cellular functions [5]. The attachment of ubiquitin to proteins is termed ubiquitination, a multistep process orchestrated by a three-enzyme cascade, wherein ubiquitin undergoes initial activation by the E1 ubiquitin-activating enzyme, is subsequently linked to the E2 ubiquitin-conjugating enzyme, and ultimately linked to target proteins via the E3 ubiquitin ligase [6]. The maintenance of cellular homeostasis depends on pathways that selectively remove abnormal, excess, or inhibitory proteins from biochemical pathways. In eukaryotic cells, this clearance is mediated by the ubiquitin–proteasome–autophagy system, in which proteins marked for degradation are tagged with ubiquitin and subsequently recognized by either the 26S proteasome or the autophagic machinery, resulting in the decomposition of the ubiquitin-tagged proteins [7].

Recent investigations have elucidated the pivotal role played by the ubiquitin-proteasome system (UPS) in tumor initiation and advancement. Dysregulation of the UPS is implicated in various processes that drive tumor progression, including cell cycle regulation, angiogenesis, proliferation, migration, invasion, and metastasis [8–10]. A study employing array-based comparative genomic hybridization [11] revealed the overexpression of E3 ubiquitin ligase MDM2 in mouse glioblastoma, where MDM2 diminishes both the levels and activity of the critical tumor suppressor p53. As a result, inactivating mutations in MDM2 may facilitate glioblastoma progression. Subsequent research into targeted therapies for MDM2 [12] led to the discovery of nutlins, a novel class of small molecules that bind directly to MDM2, preventing its interaction with p53. This interaction enhances p53 functionality, potentiates p53-dependent apoptotic pathways, and suppresses glioblastoma proliferation. Another investigation [13] identified E3 ubiquitin ligase RNF203 among the most commonly altered genes (21%) in adrenocortical carcinoma, suggesting that RNF203 functions as a negative regulatory element in WNT/*β*-catenin signaling and its possible function as a tumor-suppressing factor. Moreover, whole-exome sequencing data for OC [14] indicated that RNF43 exhibits frequent alterations in mucinous OC. Given that RNF43 downregulates WNT signaling via targeted ubiquitin-dependent degradation of Frizzled receptors, loss-of-function mutations in RNF43 might promote ovarian mucinous tumor progression. These findings highlight the promise of targeting ubiquitination-related genes (UbRGs) as a novel approach in cancer therapy.

A novel prognostic model for OC was developed by integrating transcriptome data from the Cancer Genome Atlas (TCGA) and Gene Expression Omnibus (GEO) databases with UbRG annotations from the ubiquitin and ubiquitin-like conjugation database (iUUCD). Comprehensive analyses assessed the impact of UbRGs on OC prognosis, identifying differentially expressed, prognosis-associated UbRGs with potential diagnostic and therapeutic value. The findings revealed that genes involved in ubiquitination are closely associated with OC prognosis, and the model demonstrated robust predictive power for tumor gene mutations and immune cell infiltration (ICI) within the tumor microenvironment (TME). Among these UbRGs, Ube2j1—featured in the prognostic model—was found to be downregulated in OC tissues and cell lines. This gene may inhibit OC growth, invasion, and metastasis by suppressing the JAK2/STAT3 pathway. Additionally, this pathway appears to influence the immune microenvironment of OC, modulating B cells, plasma cells, CD4+ memory T cells, M1 macrophages, and PD-L1 expression. These insights provide new perspectives for immunotherapeutic strategies targeting UbRGs in OC. In summary, this study introduces a novel prognostic model for OC and deepens our understanding of Ube2j1's role in disease progression.

## 2. Methodologies and Materials

### 2.1. Data Collection and Processing

RNA sequencing and clinical data from 414 OC tissues were procured from TCGA database (https://www.cancer.gov/ccg/research/genome-sequencing/tcga). The GSE165808 dataset was procured from the GEO database (https://www.ncbi.nlm.nih.gov/geo/). Data were processed using the R package (Version 4.4.1). The UbRGs were procured from the iUUCD repository (http://iuucd.biocuckoo.org/), recognized as a continuously evolving and comprehensive resource in this field [15].

### 2.2. Development and Evaluation of the Prognostic Risk Model

We followed the methods of Huang et al. [16]. The expression matrix for all UbRGs was derived through the limma package in R software and subsequently integrated with the clinical data from OC patients [17]. Consensus clustering of UbRGs was executed utilizing the “ConsensusClusterPlus” R package (Version 4.4.1) [18]. Two distinct molecular subtypes were ascertained by *K*-means clustering grounded in the UbRG expression profiles. Following this, univariate Cox regression analysis (UCRA) was utilized to ascertain prognostic UbRGs. Prognostic UbRGs were subjected to LASSO regression analysis utilizing the glmnet and survival packages in R software [19]. Ultimately, 22 UbRGs were chosen to develop a novel prognostic model. Ultimately, a total of 22 ubiquitously expressed genes (UbRGs) were identified as optimal candidates for constructing a novel prognostic model. Furthermore, the training dataset and external validation datasets were divided into high-risk and low-risk cohorts according to the median UbRG score. To evaluate the model's robustness and predictive capability analysis, Kaplan–Meier (K–M) survival curves and receiver operating characteristic (ROC) curves were generated.

### 2.3. Gene Mutation Analysis

Somatic mutation data for individuals with TCGA-OV were retrieved utilizing the R package “maftools” [20]. This data was subsequently analyzed to examine variations in somatic mutations between the high and low gene expression cohorts in individuals with TCGA-OV.

### 2.4. ICI Analysis and Tumor Immunoanalysis

The penetration degrees of 22 immune cell categories were examined utilizing the expression patterns of the 22 prognostic UbRGs integrated into the predictive model (model genes). A box diagram was created to examine immune checkpoint gene levels between the low- and high-risk cohorts.

### 2.5. Predictive Value of the Selected UbRGs in OC Prognosis

The CPTAC protein repository was retrieved through NCBI (https://www.ncbi.nlm.nih.gov/). The comparative protein abundance of 22 genes among normal and OC specimens in the model was examined utilizing the R package.

### 2.6. Gene Ontology (GO) and Kyoto Encyclopedia of Genes and Genomes (KEGG) Enrichment Analyses

GO enrichment analysis was conducted utilizing the EnrichGO function from the “clusterProfiler” R package. KEGG pathway analysis was executed employing the EnrichKEGG function of the same package [21].

### 2.7. Cell Culture and Cell Transfection

We followed the methods of Huang et al. [16]. The OC cell lines A2780, HEY, and SKOV3, together with the noncancerous human ovarian epithelial cell line IOSE80, were acquired from the Basic Medical Sciences Institute's Cell Center at the Chinese Academy of Medical Sciences. The specific sequences targeting Ube2j1 siRNA are depicted in Table 1.

### 2.8. Quantitative Real-Time Polymerase Chain Reaction (qRT-PCR)

Total RNA was procured from the specified cell lines employing the Total RNA Extraction Kit (Takara, Kyoto, Japan) per the supplier's protocol. Subsequently, the RNA sample underwent reverse transcription into complementary DNA with the PrimeScript RT Reagent Kit (Takara, Tokyo, Japan), and specific primers were employed for the amplification of DNA. In brief, mRNA expression levels were quantified via qRT-PCR, with each mRNA value normalized against GAPDH, which served as the endogenous control in all assays. The primer sequences are depicted in Table 2.

### 2.9. Western Blot (WB)

We followed the methods of Huang et al. [16]. The membranes were kept with primary antibodies: anti-Smu1, anti-Ube2j1 (1:1000, ABclonal, Beijing, China), and anti-GAPDH (1:5000, Bioworld, Nanjing, China).

### 2.10. Cell Proliferation

Cell viability was ascertained utilizing the cell counting kit-8 (CCK-8) assay (MCE, South Brunswick Township, NJ, United States). Briefly, the cells were inoculated in a 96-well plate at a 1000 cells per well, measuring cell viability for Days 0, 1, and 2 according to the manufacturer's instructions.

### 2.11. Transwell Assay and Wound Healing Assay

The invasion and migration capabilities of cells were assessed using 24-well transwell inserts (8.0 *μ*m, BD Biosciences, Bedford, MA, United States). For the migration assay, 4 × 10^5^ cells were seeded into the upper chamber, while 600 *μ*L of medium containing 10% FBS was added to the lower chamber of the 24-well plate. In the invasion assay, the inserts were first precoated by diluting the substrate matrix with basal medium at a ratio of 1:8. Then, 80 *μ*L of the diluted matrix was applied to the upper chambers. The subsequent procedures mirrored those of the migration assay, with 5 × 10^5^ cells seeded. The cells were incubated at 37°C for 72 h, after which the invaded or migrated cells were fixed, stained, and counted.

For the wound healing assay, 2 × 10^6^ cells were placed in a 12-well plate. Following a 24-h incubation, a wound was introduced mechanically using a sterile 200 *μ*L pipette tip. Cell migration was detected by photomicrography at 0, 48, and 72 h utilizing a phase contrast microscope.

### 2.12. Immunohistochemistry (IHC)

We followed the methods of Huang et al. [16]. The levels of Ube2j1 within clinical specimens were detected by IHC utilizing an anti-Ube2j1 antibody (1:200, ABclonal, Beijing, China). ImageJ software facilitated integrated optical density (IOD) measurements [22]. Data were denoted as average optical density, calculated by the following formula: IOD/area.

### 2.13. Statistical Analysis

Data analysis and the generation of statistical charts were executed utilizing R software (Version 4.4.1, New York, NY, United States), ImageJ, and GraphPad Prism 9. All measurement data are denoted as means ± SD (*n* = 3 unless stated otherwise). The comparison between pairs of cohorts employed a *t*-test, whereas multiple group comparisons were executed utilizing a one-way analysis of variance (ANOVA). Statistical significance was defined as *p* < 0.05.

## 3. Results

### 3.1. Prediction Model Development

**Figure 1:**
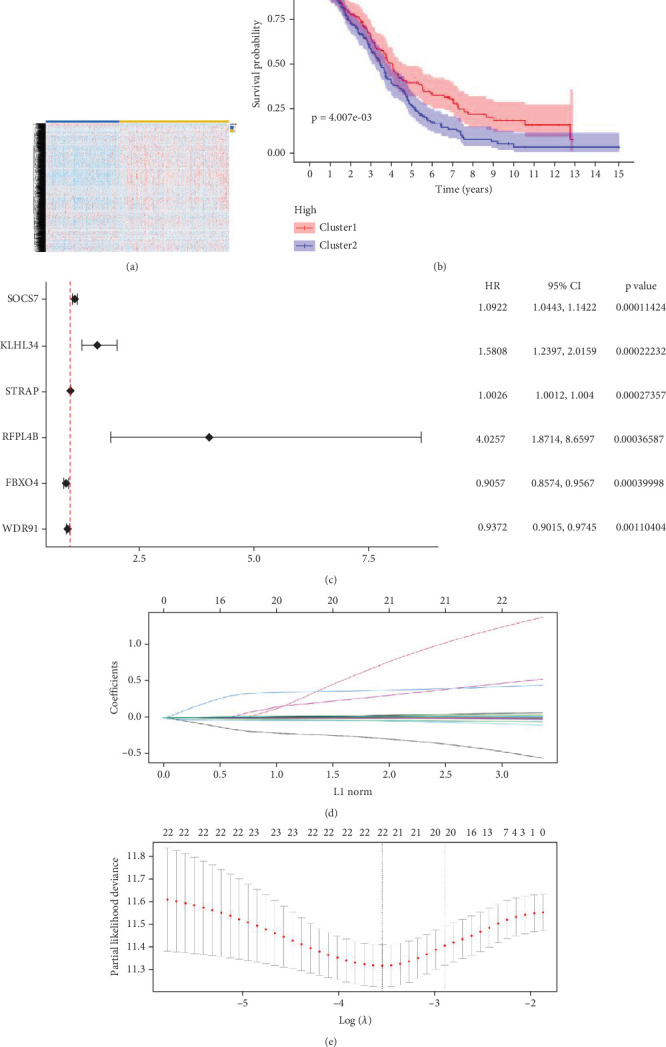
Screening and categorization of OS-related UbRGs in OC. (a) A heat map depicting the unsupervised clustering of 414 individuals with TCGA-OV into two distinct cohorts grounded in the high and low expression of all UbRGs within TCGA-OV datasets is shown. (b) K–M survival curves (log-rank test, *p* < 0.01) illustrating OS for Clusters 1 and 2 are presented. (c) Through COX univariate analysis, 23 UbRGs were identified as being markedly linked to prognosis (*p* < 0.01). (d) The allocation of LASSO coefficients for the 23 prognosis-related UbRGs is displayed. (e) A coefficient profile plot was generated against the log (lambda) sequence in the LASSO model, with the optimal lambda parameter denoted by the first black dotted line.

The expression data of 929 UbRGs from the iUUCD were procured from TCGA-OV dataset. Unsupervised clustering analysis and principal component analysis (PCA) were utilized to classify individuals with TCGA-OV into two distinct clusters grounded in the expression patterns of these UbRGs (Figure 1a and Figure S1A). The K–M survival curves suggested notable differences in survival outcomes between the two clusters (log-rank test, *p* < 0.01) (Figure 1b). Using UCRA, which was based on the association between UbRGs and overall survival (OS) of individuals with OC in TCGA database, 23 prognosis-related genes were identified: ASB2, DMWD, EML2, FBXO4, KCTD11, KLHL34, KMT2B, RFPL4B, RNF144B, RNF175, SKP2, SMU1, SOCS7, STRAP, TLE2, TRAF4, TRIM27, TRIM69, UBE2J1, UBE2Q2, WDR91, WDTC1, and ZBTB4 (Figure 1c and Table S1D, *p* < 0.01). Afterward, LASSO regression analysis was executed on these 23 UbRGs to establish a prognostic model for OC, with regression coefficient calculations displayed in Figure 1d. We further assessed the partial likelihood deviance of these 23 UbRGs as they were incorporated into the model. Upon incrementally reducing the number of genes and analyzing the changes in partial likelihood deviance with increasing log (lambda) values, it was observed that the deviance reached its minimum when 22 genes were included. This finding indicated that the model achieved optimal performance when 22 UbRGs (with TLE2 excluded) were incorporated (Figure 1e). Furthermore, GO and KEGG enrichment analyses of these 22 genes within TCGA-OV dataset revealed significant enrichment in functions associated with ubiquitination (Figure S1B, S1C).

### 3.2. Prognostic Prediction Potential of 22 UbRGs in OC

**Figure 2:**
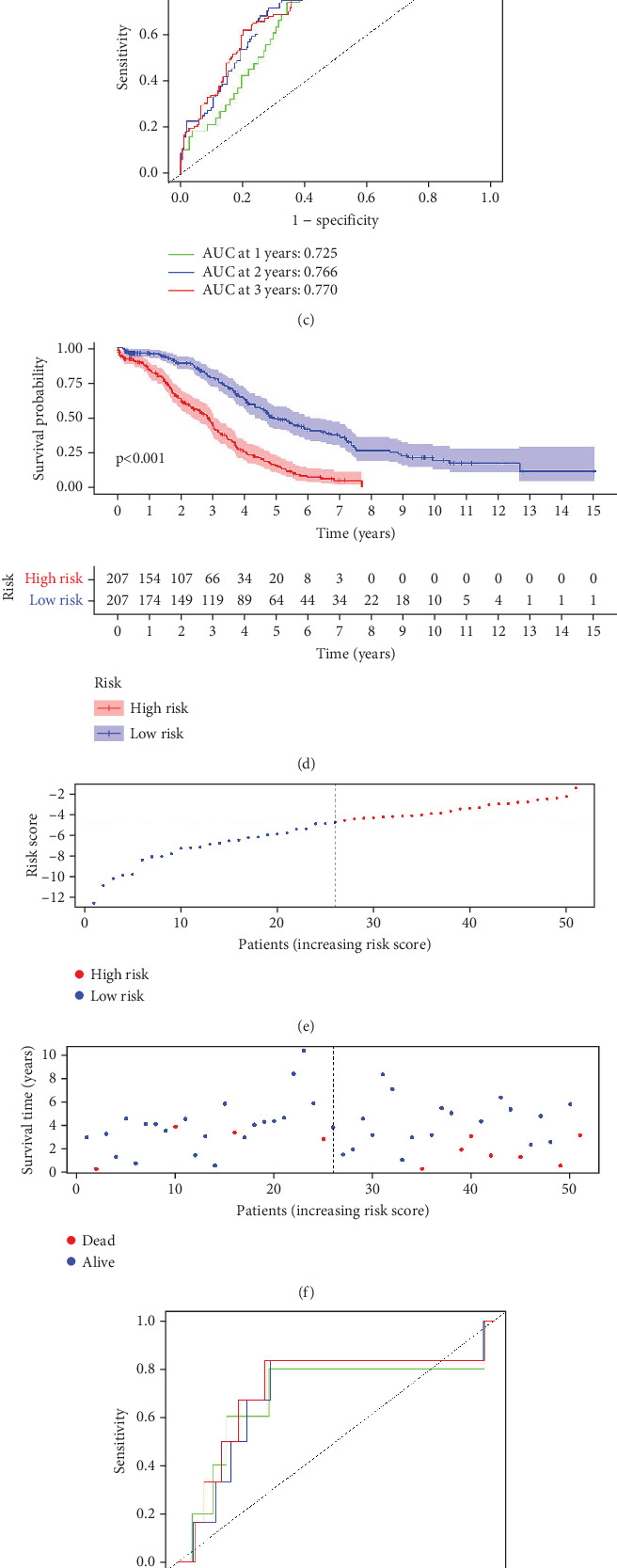
Screening and verification of OS-related UbRGs in individuals with OV. (a) Risk score allocation among individuals with TCGA-OV datasets, with red and blue represent the high-risk and low-risk groups, respectively. (b) Different survival statuses of high- and low-risk individuals in TCGA-OV datasets, with red and blue represent dead and living ones, respectively. (c) The ROC curves of 1, 3, and 5 years of the optimized 22 genes in the training set. (d) K–M survival curves for the low- and high-risk cohorts in the training set (*p* < 0.01). (e) Risk score allocation in individuals in the GSE165808 datasets, with red and blue represent the high-risk and low-risk groups, respectively. (f) Different survival statuses of high- and low-risk individuals in the GSE165808 datasets, with red and blue represent dead and living ones, respectively. (g) The ROC curves of 1, 3, and 5 years of the optimized 22 genes in the test set. (h) K–M survival curves for the low- and high-risk cohorts in the test set (*p* < 0.05).

Utilizing the 22 UbRGs chosen for developing the prognostic model, specimens in the total dataset were stratified into high-risk and low-risk cohorts per the median risk score. The risk curves for the high-risk and low-risk cohorts are depicted in Figure 2a,b. The constructed prediction model was employed to calculate risk scores for TCGA samples, thereby categorizing them into high- and low-risk groups based on the median risk score. As expected, the high-risk cohort exhibited significantly higher risk scores than the low-risk cohort. Correspondingly, the high-risk group demonstrated a markedly higher mortality rate when compared to the low-risk group.

To further assess the predictive accuracy, multi-index ROC curves were generated, as illustrated in Figure 2c. The area under the curves (AUCs) for 1-, 2-, and 3-year survival were 0.725, 0.766, and 0.770, respectively, indicating moderate predictive accuracy. Additionally, survival curves for both cohorts are plotted in Figure 2d, revealing markedly lower survival rates in the high-risk cohort compared to the low-risk cohort. Consistently, analogous results were derived from the external validation dataset. In this dataset, samples were likewise segregated into high- and low-risk cohorts based on the median risk score. Based on the established prediction model, we conducted risk scores on 51 samples in the GSE165808 datasets. As shown in the risk curve in Figure 2e, after grouping according to the median, it was found that there were 26 patients in the low-risk group and 25 patients in the high-risk group. We further analyzed the number of deceased and surviving patients in the two groups. As shown in Figure 2f, it was found that the proportion of deaths in the low-risk group was 4/26, while that in the high-risk group was 7/25. It showed that the mortality rate of patients from the low-risk group to the high-risk group gradually increased. Multi-index ROC curves were again plotted to evaluate survival curve accuracy, as presented in Figure 2g. The AUCs for 1-, 2-, and 3-year survival were 0.689, 0.701, and 0.718, respectively, reflecting moderate predictive capability. Finally, survival curves for the high-risk and low-risk cohorts were generated, as depicted in Figure 2h, showing markedly diminished survival rates in the high-risk cohort relative to the low-risk cohort.

### 3.3. Relationship Between Prediction Model and Gene Mutations

**Figure 3:**
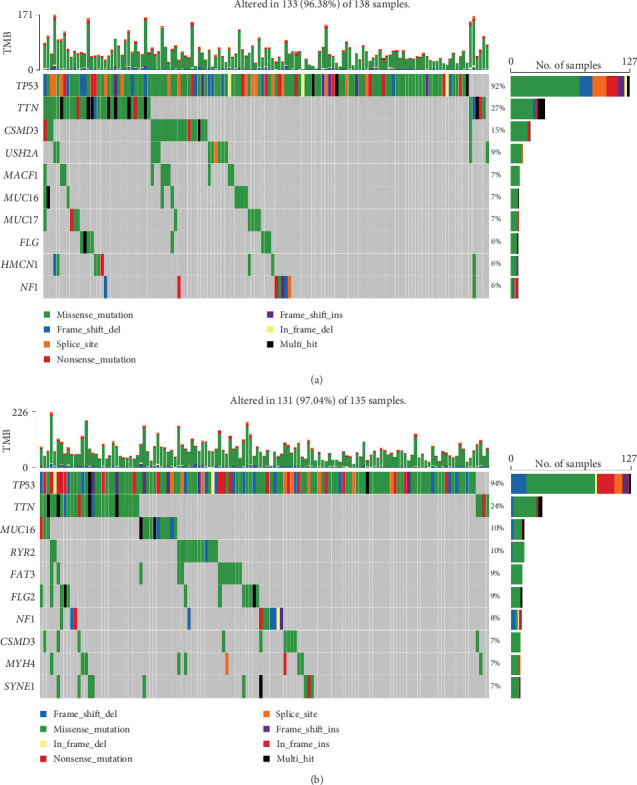
The somatic mutation type of mutation variation in the high- and low-risk cohorts. (a) The Top 10 mutation genes in the high-risk cohort and the corresponding mutation types and proportions. (b) The Top 10 mutation genes in low-risk cohort and the corresponding mutation types and proportions.

After risk stratification of TCGA-OV patients based on the prediction model, analysis of gene mutation types revealed significant mutations in TP53 (92%), TTN (27%), CSMD3 (15%), USH2A (9%), MACF1 (7%), MUC16 (7%), MUC17 (7%), FLG (6%), HMCN1 (6%), and NF1 (6%) in the high-risk cohort. In the low-risk cohort, significant mutations were observed in TP53 (94%), TTN (24%), MUC16 (10%), RYR2 (10%), FAT3 (9%), FLG2 (9%), NF1 (8%), CSMD3 (7%), MYH4 (7%), and SYNE1 (7%) (Figure 3a,b).

### 3.4. Relationship Between Prediction Models and Immunity

**Figure 4:**
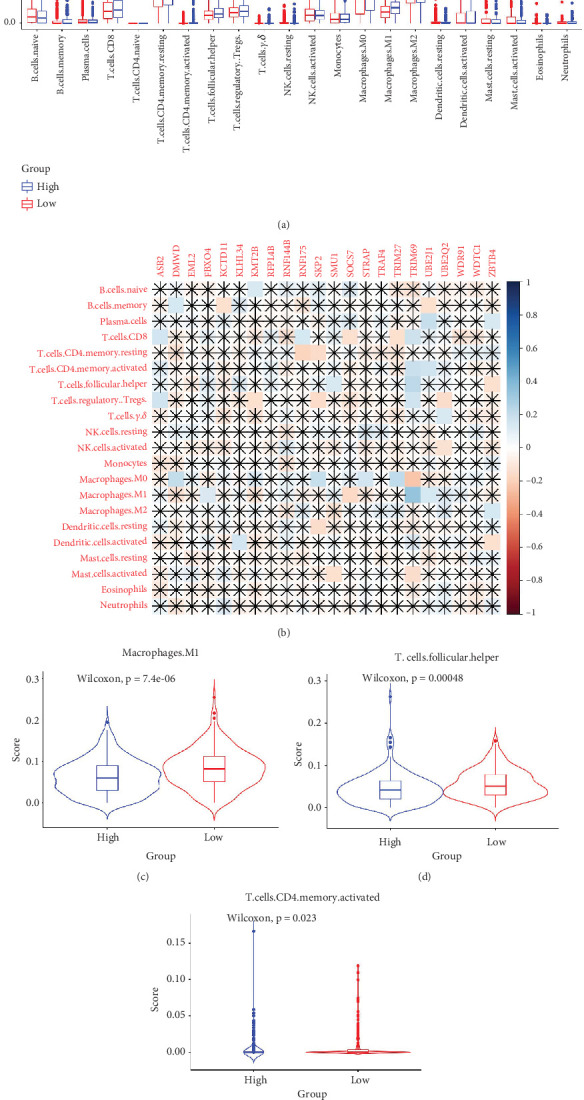
Immune characterization of prognostic UbRGs in the OV microenvironment. (a) A histogram illustrating differential ICI, with red and blue bars representing the high- and low-risk cohorts, respectively. (b) The link between the expression of 22 prognostic-related UbRGs and the distribution of 22 infiltrating immune cell subsets was assessed utilizing the CIBERSORT method. The asterisk indicates that there is no obvious correlation between the gene corresponding to the point and the immune cell. Blue indicates a positive correlation between the two, and yellow indicates a negative correlation. (c) A box plot demonstrating the variation in infiltration of activated CD4 memory T cells between the high- and low-risk cohorts (*p* < 0.05). (d) A box plot revealing the variations in follicular helper T cell infiltration between the high- and low-risk cohorts (*p* < 0.05). (e) A box plot displaying the contrasting levels of M1 macrophage infiltration observed between the high- and low-risk cohorts (*p* < 0.05).

The TME encompasses diverse cell types, including tumor cells, stromal cells (such as fibroblasts and endothelial cells), different immune cells (such as T lymphocytes, NK cells, macrophages, and dendritic cells), and extracellular matrix components (e.g., biochemical factors secreted by these cells) [23]. These cellular elements engage in mutual interactions and collectively influence tumor behavior, determining whether the tumor undergoes regression or progression. The TME serves a pivotal function not only in tumor growth, invasion, and metastasis but also in determining the response to treatment [22]. Advances in high-throughput sequencing technology and machine learning have led to the development of cell-type identification by estimating relative subsets of RNA transcripts (CIBERSORT), a tool designed to infer the composition of stromal and immune cells in tumors based on RNA sequencing data [24]. By applying the CIBERSORT algorithm, distinctions in ICI between high-risk and low-risk cohorts were evaluated. It was noted that the high-risk cohort exhibited markedly elevated frequencies of activated CD4 memory T cells, T follicular helper cells, and M1 macrophages relative to the low-risk cohort (Figures 4a, 4c, 4d, and 4e). These observations suggest that low-risk patients experience stronger immune responses within the tumor environment than their high-risk counterparts. The infiltration of these immune cells may have a substantial impact on OC patient outcomes. Furthermore, the links between the 22 UbRGs and ICI were examined using the CIBERSORT algorithm. The analysis revealed that most of these genes modulated the TME in OC. The asterisk indicates that there is no obvious correlation between the gene corresponding to the point and the immune cell. Blue indicates a positive correlation between the two, and yellow indicates a negative correlation. TRIM69, Ube2j1, ZBTB4, and SKP2 show the most pronounced effects on the immune microenvironment of the tumor (Figure 4b). In summary, these results indicate that relative to the high-risk cohort, the low-risk cohort displayed more robust immune responses, which may enhance the potential for immunotherapy. Moreover, the differential expression of TRIM69, Ube2j1, ZBTB4, and SKP2 likely serves a critical function in modulating tumor immune infiltration.

### 3.5. Gene Expression Prediction Model and Screening

**Figure 5:**
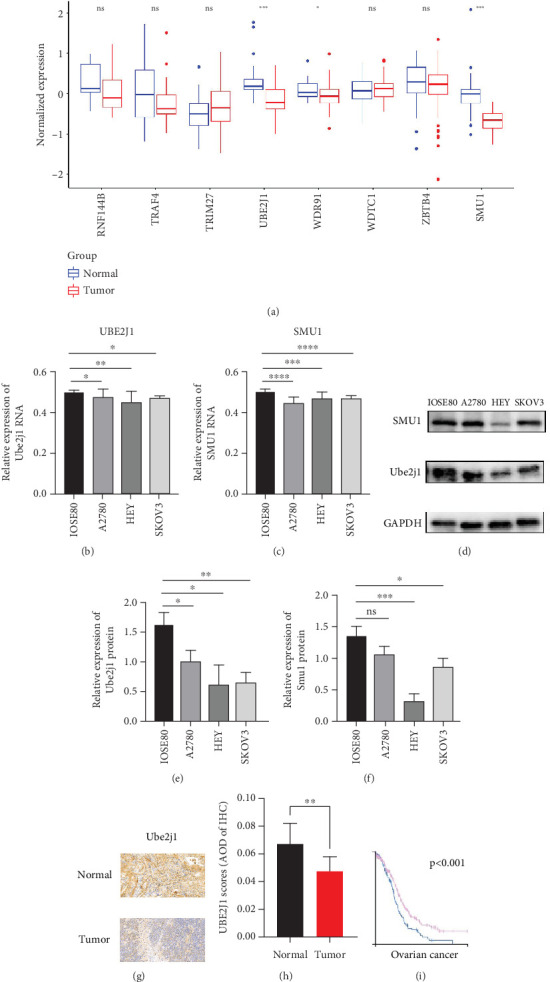
Expression of prognosis-related UbRGs in OV. (a) Box diagrams illustrating the protein expression levels of prognostic-related UbRGs in CPTAC pairs. (b, c) qRT-PCR was executed to evaluate the mRNA levels of Ube2j1 and Smu1 in three OC cell lines and one normal ovarian epithelial cell line. (d–f) WB analysis was performed to evaluate the protein expression levels of Ube2j1 and Smu1 in three OC cell lines and one normal ovarian epithelial cell line. (g, h) IHC analysis was employed to compare the Ube2j1 expression between OC tissues and normal ovarian tissues. (i) K–M plots revealed that elevated Ube2j1 levels are markedly linked to improve patient survival (*p* < 0.001).

As UbRGs exert their functions at the protein level, the CPTAC protein expression database was employed to analyze the differential protein expression of prognostic-related UbRGs in OC. The analysis revealed significant downregulation of Ube2j1, SMU1, and WDR91 in OC tissues (Figure 5a). Among these, Ube2j1 and SMU1 exhibited the most prominent differences between normal ovarian tissues and OC. The mRNA expression of these two prognostic-associated UbRGs was then evaluated in three OC cell lines (A2780, HEY, and SKOV3) and a normal ovarian epithelial cell line (IOSE80). The mRNA levels of both Ube2j1 (Figure 5b) and SMU1 (Figure 5c) were markedly diminished in all three OC cell lines in contrast to IOSE80 cells. WB analysis demonstrated a significant decrease in Ube2j1 protein expression (Figure 5d,e) across all three OC cell lines, whereas SMU1 protein expression (Figure 5d,f) was only diminished in HEY and SKOV3 cells relative to IOSE80 cells, with no notable changes observed in A2780 cells. To further validate these findings, clinical specimens (comprising three OC and three normal ovarian tissues) were procured, and IHC analysis showed reduced Ube2j1 protein expression in OC tissues relative to normal ovarian tissues (Figure 5g,h). Furthermore, K–M survival curves from the Human Protein Atlas (HPA) database indicated that low Ube2j1 levels in OC correlated with poor prognosis (Figure 5i). Collectively, these observations indicate that Ube2j1 serves as a critical prognostic-related gene in OC. Consequently, the biological functions of Ube2j1 in OC progression were further investigated in subsequent experiments.

### 3.6. Knockdown of UBE2J1 Stimulates Proliferation, Invasion, and Migration of OC Cells

**Figure 6:**
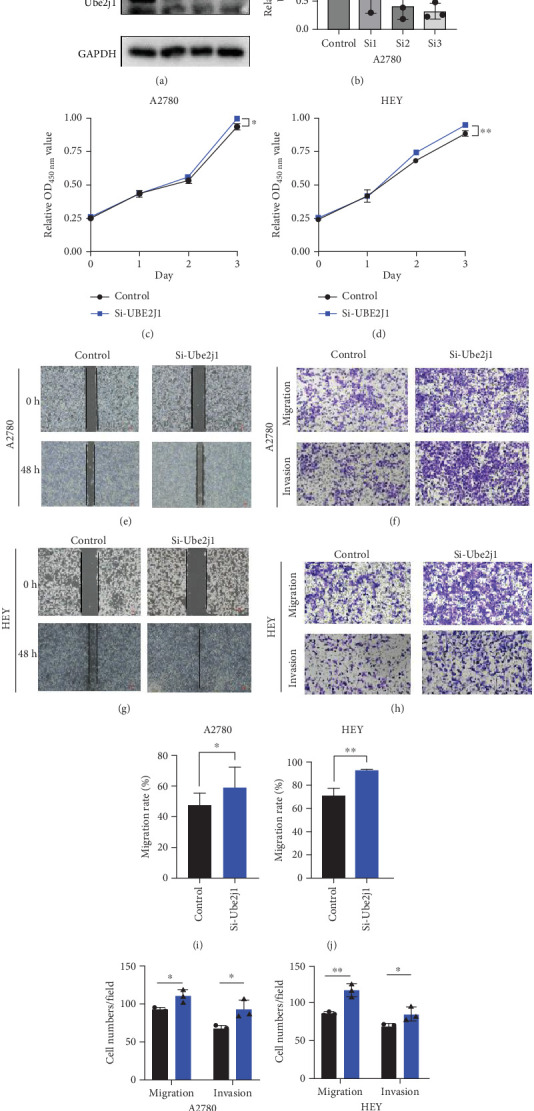
Ube2j1 influences the proliferation, invasion, and migration capabilities of OV cells. (a, b) WB was conducted to assess the knockdown effectiveness of Ube2j1 in A2780 cells. (c, d) CCK-8 assay was utilized to evaluate the proliferative capacity (OD450) of A2780 and HEY cells following Ube2j1 knockdown. (e, i) Wound healing assay was employed to investigate the migratory capability of A2780 cells post-Ube2j1 knockdown (⁣^∗^*p* < 0.05; ⁣^∗∗^*p* < 0.01). (g, j) Wound healing assay was carried out to evaluate the migratory capacity of HEY cells after Ube2j1 knockdown (⁣^∗^*p* < 0.05; ⁣^∗∗^*p* < 0.01). (f, k) Transwell assay was executed to assess the invasive and migratory capabilities of A2780 cells following Ube2j1 knockdown (⁣^∗^*p* < 0.05; ⁣^∗∗^*p* < 0.01). (h, l) Transwell assay was conducted to evaluate the invasive and migratory capabilities of HEY cells post-Ube2j1 knockdown (⁣^∗^*p* < 0.05; ⁣^∗∗^*p* < 0.01).

To investigate the role of Ube2j1 in the malignant progression of OC, A2780 cells were transiently transfected with siRNA targeting Ube2j1. After confirming efficient knockdown of Ube2j1 (Figure 6a,b), the proliferative, invasive, and migratory properties of OC cells (A2780 and HEY) were assessed. Results from the CCK-8 assay revealed that silencing Ube2j1 led to increased cell proliferation (Figure 6c,d). Additionally, wound healing and invasion–migration assays demonstrated enhanced wound closure and elevated invasion and migration capabilities in Ube2j1-silenced cells (Figures 6e, 6f, 6g, 6h, 6i, 6j, 6k, and 6l). These findings highlight the critical role of Ube2j1 in suppressing OC malignancy.

### 3.7. UBE2J1 Negatively Regulates the JAK2/STAT3/PD-L1 Signaling Pathway, Promotes Immune Remodeling, and Inhibits OC Progression

**Figure 7:**
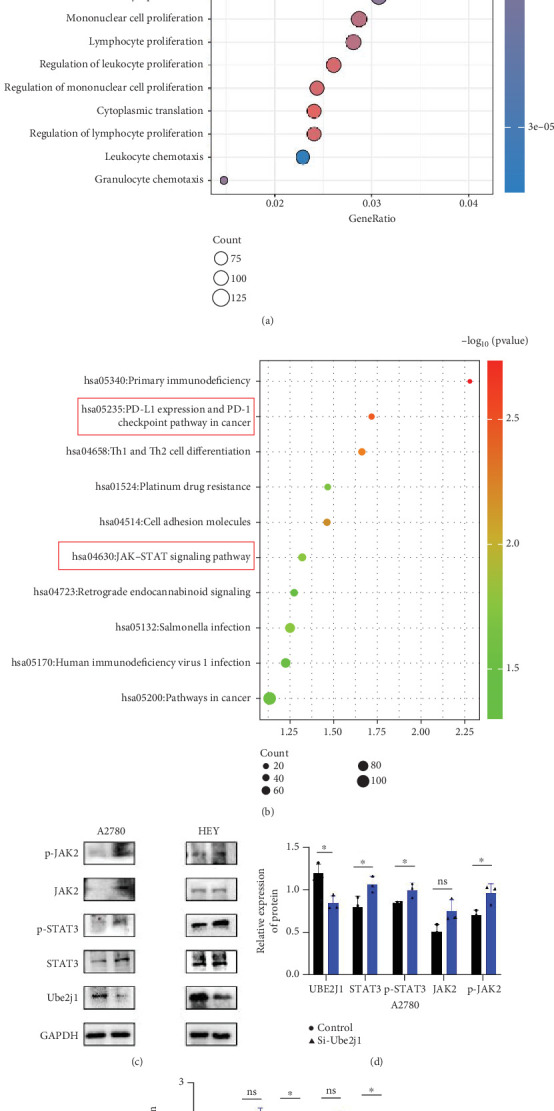
Knocking down Ube2j1 in A2780 and HEY cells activated the JAK2/STAT3 signaling pathway. (a) Bar plot is denoting enriched functions of the upregulated genes coexpressed with Ube2j1. (b) Bar plot is denoting enriched pathways of the upregulated genes coexpressed with Ube2j1. (c–e) WB was performed to detect that knocking down Ube2j1 in A2780, and HEY cells activated the JAK2/STAT3 signaling pathway.

To explore how Ube2j1 influences OC malignancy progression, differential expression genes were examined based on Ube2j1 expression levels in TCGA-OV patients through GO and KEGG enrichment analyses. GO enrichment analysis suggested significant associations with immune response, monocyte differentiation, and T cell activation (Figure 7a). KEGG enrichment analysis identified pathways related to the JAK/STAT signaling cascade, which has been implicated in promoting cancer progression by enhancing PD-L1 expression and altering immune infiltration profiles. Notably, this analysis also highlighted the PD-1/PD-L1 signaling pathway (Figure 7b). Further validation by WB of the JAK/STAT pathway demonstrated that Ube2j1 knockdown activated the JAK2/STAT3 signaling axis in two OC cell lines (Figures 7c, 7d, and 7e). In conclusion, Ube2j1 is diminished in individuals with OC and cell lines and may suppress OC cell proliferation, invasion, and metastasis via suppression of the JAK2/STAT3 pathway. This pathway potentially modulates PD-L1 expression and reshapes the immune microenvironment of OC (including alterations in B cells, plasma cells, memory CD4 T cells, and M1 macrophages), thereby increasing the sensitivity of patients to immunotherapy agents, such as ICIs. These findings offer novel insights into UbRG-based immunotherapy for OC patients.

## 4. Discussion

Ubiquitination has been identified to serve a pivotal function in controlling essential cancer characteristics, encompassing the evasion of growth suppressors, reprogramming of energy metabolism, unlocking of phenotypic plasticity, modulation of the microbiome, and the behavior of senescent cells [25, 26]. In recent years, a growing body of literature has introduced novel strategies for targeting ubiquitination pathways in cancer therapy. These strategies primarily involve the development of inhibitors that block the molecular connections between E1 and E2 enzymes or between E2 and E3 ligases. One such example is Leucetta A, which has been demonstrated to impede the binding between ubiquitin-conjugating enzyme 13 and ubiquitin-conjugating enzyme variant 1A, effectively preventing the formation of their complex [27]. In addition, compounds such as manadosterols A and B, derived from the sponge *Lissodendoryx fibrosa*, have been found to obstruct the Ubc13-Uev1A interaction, thereby inhibiting the assembly of the Ubc13-UEV1A complex and preventing the formation of K63-linked polyubiquitin chains, which ultimately results in the suppression of substrate degradation [28]. Moreover, the inhibitor CC0651, targeting the E2 enzyme Cdc34, prevents the ubiquitination and degradation of p27, thus hindering tumor cell proliferation [29]. Among the representative drugs aimed at targeting E3 ligases are PROTACs and molecular glues, with several of these therapies currently in Phase I–II clinical trials, encompassing ARV-110 and ARV-47, both in Phase II trials [30], and CC-90009, which is undergoing Phase II trials for leukemia treatment [31]. These developments indicate that UbRGs might serve as a potential therapeutic target in cancer interventions. Given the essential role of protein ubiquitination in regulating biological functions and its significant involvement in disease progression [32, 33], particularly in the context of tumors, exploring the therapeutic potential of UbRGs in OC could contribute to advancing diagnosis and treatment strategies for this disease.

This study investigated the association between UbRGs and OC. Examination of TCGA-OV dataset indicated that patients could be categorized into two distinct cohorts based on UbRG expression, which were linked to OC prognosis. A set of 22 UbRGs was subsequently identified and utilized to construct a prognostic model. In addition, a nomogram incorporating the UbRG score and clinicopathological characteristics was developed to estimate OS in OC patients. Calibration curves suggested that the nomogram exhibited robust forecasting power, particularly for long-term survival. Both the training and testing cohorts confirmed that the prognostic model provided accurate and reliable predictions. These findings indicate that the UbRG score exhibits significant links to OC advancement and outcome.

To delve deeper into the relationship between UbRGs and their molecular features in OC, patients in TCGA-OV cohort were categorized into high-risk and low-risk groups based on the prognostic model. Notable distinctions were identified between these groups, particularly in terms of gene mutation profiles, ICI, and molecular function characteristics.

Genomic instability, defined by elevated mutation frequencies in cell lines, is regarded as a fundamental characteristic of carcinogenesis in multicellular organisms [34, 35]. It has been reported that ubiquitin-specific protease (USP) serves a function in the regulation of genomic instability and gene mutations. For example, the E3 ligase RNF126 ubiquitinates MRE11, thereby activating the DNA damage response in triple-negative breast cancer and contributing to resistance to radiotherapy [36]. Furthermore, ataxia telangiectasia mutated (ATM), an essential kinase responding to double-strand breaks (DSBs) [37], has been shown to phosphorylate and activate the E3 ubiquitin ligase Peli1, promoting the ubiquitination of NBS1 and facilitating the recruitment of the ATM and MRN complex at DSB sites [38, 39]. With regard to gene mutations in this study, notable distinctions emerged when comparing the high-risk and low-risk cohorts: CSMD3, USH2A, and MACF1 alterations exhibited elevated occurrence in the high-risk cohort, while MUC16, RYR2, and FAT3 modifications appeared more frequently in the low-risk cohort. Certain genes may function as tumor suppressors or promoters in normal tissues, but their roles might be reversed in tumor tissues due to changes in other genes or environmental factors, thus either promoting or inhibiting tumor progression [40]. Indeed, it has been reported that mutations in CSMD3 are strongly associated with increased tumor mutational burden in OC and poor clinical prognosis [41]. Additionally, research has demonstrated that MUC16 interacts with Siglec-9 on neutrophils, leading to inflammation and immunosuppression in OC [42]. These findings suggest that USP may regulate these gene mutations, suppressing their functions and altering their impact on OC malignant behavior and the TME. This indicates that UbRGs likely influence OC malignant behavior by modulating gene mutation patterns.

Tumors are intricately linked to and constantly interact with their surrounding microenvironment. Tumor-infiltrating immune cells, which represent crucial elements of the TME, function as independent predictors of tumor survival. The interactions between immune cells and cancer cells within the TME are pivotal for tumor advancement and therapeutic resistance development [43–46]. It has been reported that USP serves an essential function in regulating TME cellular signaling pathways [47]. This modification effectively enhances antitumor immunity and maintains the balance between tumor suppressors and oncoproteins through the modulation of immune responses [48]. The UPS can also impact the TME by regulating, both directly and indirectly, the breakdown of immune checkpoint proteins and the secretion of cancer-promoting cytokines [49]. To assess the influence of 22 prognostic UbRGs on the immune microenvironment of OC, patients were categorized into high-risk and low-risk cohorts grounded in the median UbRG scores. The CIBERSORT algorithm was then applied to ascertain the link between these UbRGs and ICI. The outcomes demonstrated a notable connection between UbRG expression and various immune cell populations. Notably, UbRG expression was associated with the prevalence of activated CD4 memory T cells, follicular helper T cells, and M1 macrophages, which are known to drive persistent and effective antitumor immune responses [1, 50–55]. The abundance of these immune cells was notably lower in high-risk patients, indicating a weaker immune response and poorer prognosis in this cohort. Furthermore, the investigation of the regulatory effects of these 22 genes on the OC TME revealed that most of the genes modulated the OC TME to varying extents, with the particular influence exerted by TRIM69, Ube2j1, ZBTB4, and SKP2.

Substantial variations in the expression of 22 UbRGs between OC and normal ovarian tissues were identified through a query of the CPTAC protein database. Ube2j1, WDR91, and SMU1 were notably downregulated in OC, with Ube2j1 and SMU1 displaying more substantial variations between normal and OC tissues. The mRNA and protein expression levels of Ube2j1 and SMU1 were then assessed in three OC cell lines (A2780, HEY, and SKOV3) and one normal ovarian cell line (IOSE80). Both Ube2j1 and SMU1 mRNA levels were notably reduced in all three OC cell lines in contrast to the IOSE80 cells. Ube2j1 protein expression was markedly diminished in all three OC cell lines, whereas SMU1 protein expression was reduced only in the HEY and SKOV3 cell lines relative to IOSE80 cells. These findings suggest that the E2 ubiquitin-conjugating enzyme Ube2j1 is pivotal in modulating OC development and progression. E2 enzymes serve a central function in the ubiquitination cascade by catalyzing the attachment of ubiquitin, which largely determines the types of chain linkages and, consequently, the type of signal and fate conferred on the modified proteins [56]. Previous studies have shown that Ube2j1 can mediate the ubiquitination and degradation of various substrate proteins, thereby regulating several cellular processes [1, 57–61]. Moreover, recent research has indicated that UBE2J1 can act as a tumor suppressor, inhibiting the proliferation and metastasis of colorectal cancer [62]. Despite these findings, the role of UBE2J1 as a ubiquitin-conjugating enzyme in cancer remains insufficiently studied. Through IHC analysis of Ube2j1 protein levels in OC tissues and K–M survival curve analysis using the HPA database, it was suggested that Ube2j1 levels was notably reduced in OC tissues and strongly associated with poor patient prognosis. Lastly, the biological roles of Ube2j1 in the malignant advancement of OC were preliminarily investigated. The invasion, migration, and proliferation abilities of A2780 and HEY cells with Ube2j1 knockdown were markedly enhanced relative to the control cohort, further indicating that Ube2j1 serves a critical regulatory function in OC progression.

To gain a deeper understanding of Ube2j1's role in OC, individuals from TCGA-OV cohort were grouped into high- and low-expression categories based on Ube2j1 levels. Subsequent GO and KEGG enrichment analyses of differentially expressed genes revealed insightful findings. GO analysis indicated a strong association with monocyte differentiation, T cell activation, and immune response modulation, consistent with the immune infiltration patterns observed in the earlier prognostic model. Ube2j1 was shown to enhance the polarization of M1 macrophages and activation of CD4 memory T cells while also impacting the regulation of other immune cell populations such as B cells and plasma cells. KEGG pathway analysis highlighted the PD-1/PD-L1 immune checkpoint and JAK/STAT signaling pathways as key mechanisms. Together, these results underscore the significant connection between Ube2j1 and the TME in OC.

Immunotherapy has shown substantial efficacy in the clinical management of various malignancies, with immune checkpoints identified as key therapeutic targets. Prior investigations have indicated that the UPS serves a vital function in regulating PD-1/PD-L1 protein levels within the TME, thereby enhancing the effectiveness of immunotherapy [49]. For instance, the SPOP protein has participated in controlling several cancer-related substrates and is essential for mediating PD-L1 degradation [63]. Furthermore, F-box proteins, as subunits of the SCF E3 ligase complex, perform various functions in human tumors, including the mediation of PD-1 ubiquitination [64]. Another study demonstrated that FBXO22 can activate PD-L1 ubiquitination, thereby increasing the sensitivity of non–small cell lung cancer cells to DNA damage [65]. The involvement of UPS in immune checkpoint regulation suggests that USP may hold potential as a novel therapeutic strategy to enhance antitumor immunity. Additionally, the stimulation of the JAK/STAT3 signaling cascade exhibits strong links to PD-L1 expression: blocking JAK/STAT3 activation not only markedly diminishes the effects of M1-like tumor-associated macrophages on OSCC cell colony formation, invasion, migration, microsphere formation, and xenograft development [66] but also inhibits PD-L1 expression on M1 macrophages, promoting their polarization and enhancing proinflammatory cytokine production [67]. Based on these findings, along with the bioinformatics analysis results, it can be inferred that Ube2j1 likely remodels the immune microenvironment of OC (particularly influencing M1 macrophage polarization) and regulates malignant behavior in OC through the JAK2/STAT3/PD-L1 pathway. Indeed, WB analysis revealed that Ube2j1 knockdown activated the JAK2/STAT3 signaling pathway in both OC cell lines.

In conclusion, UbRGs have been identified as effective prognostic indicators for OC, exhibiting strong associations with the regulation of the immune microenvironment. Notably, Ube2j1, a pivotal enzyme in the ubiquitination cascade, demonstrates considerable impact on controlling malignant behavior and ICI in OC, highlighting its potential as both a therapeutic target and a prognostic marker. This study explored the relationship between UbRGs and OC, demonstrating that the prognostic model correlates robustly with gene mutations and immune infiltration, thus highlighting the essential function of UbRGs in determining outcomes and therapeutic approaches for OC. Specifically, the involvement of Ube2j1, an E2 ubiquitin-conjugating enzyme, was confirmed in OC, revealing its close association with immune infiltration. Furthermore, the mechanism by which Ube2j1 modulates the OC phenotype and reshapes its immune microenvironment via the JAK2/STAT3/PD-L1 pathway was elucidated, providing novel insights into the potential for ubiquitination-based immunotherapy in OC.

## 5. Conclusion

In this investigation, the expression profiles of UbRGs in OC were comprehensively analyzed, leading to the identification and characterization of two distinct molecular subtypes based on these expression patterns. Moreover, a UbRG score was constructed, demonstrating that OC patients with lower UbRG scores exhibited a more favorable prognosis. Upon evaluating potential biomarkers for immunotherapy, it was observed that patients with elevated UbRG scores could derive greater benefit from such treatments. These discoveries markedly advance our knowledge of the TME and ICI in OC, offering potential guidance for establishing precise and potent immunotherapeutic approaches. Particularly, Ube2j1, a key UbRG, emerged as a prospective indicator and therapeutic target in OC. The mechanism by which Ube2j1 influences OC phenotype and modulates the immune microenvironment through the JAK2/STAT3/PD-L1 signaling pathway was elucidated, providing novel insights for the application of ubiquitination-based immunotherapy in OC.

## Figures and Tables

**Table 1:** Three target sequences of Ube2j1 small interfering RNA (siRNA).

	**Sense (5**⁣′**-3**⁣′**)**	**Antisense (5**⁣′**-3**⁣′**)**
siUbe2j1-1	GCUCUUAUAUUCCGACGAAUATT	UAUUGGUCGGAAUAUAAGAGCTT
siUbe2j1-2	CCACCAAGCAUUAUUCUCCUATT	UAGGAGAAUAAUGCUUGGUGGTT
siUbe2j1-3	CGUGGAGUAUAAGGACAGCAUTT	AUGCUGUCCUUAUACUCCACGTT

**Table 2:** The primer sequences utilized in the qRT-PCR experiments.

	**Sense (5**⁣′**-3**⁣′**)**	**Antisense (5**⁣′**-3**⁣′**)**
SMU1	ACAAACACAGCCAGAGCGAT	TTCCATCTGGGTATGCCTCAC
Ube2j1	GAGACCCGCTACAACCTGAAG	CGCATGGTAATGATCTGTTGGAT
GAPDH	CGGAGTCAACGGATTTGGTCGTAT	AGCCTTCTCCATGGTGGTGAAGAC

## Data Availability

The data used to support the findings of this study are included within the article.

## Supporting Information

Supporting Information Additional supporting information can be found online in the Supporting Information section. Figure S1: Screening and categorization of OS-related UbRGs in OC. (S1A) PCA suggested that the UbRG levels were able to distinctly separate individuals with TCGA-OV into two cohorts: Cohort 1 and Cohort 2. (S1B) GO enrichment analysis was conducted on key prognostic genes. (S1C) KEGG pathways for these key prognostic genes were identified. Figure S2: Experimental flow chart. Table S1: Reagents are mainly used.
