# Correction to “Prognostic Value of Ubiquitination-Related Genes in Ovarian Cancer and Their Correlation With Tumor Immunity”

**DOI:** 10.1155/humu/9879845

**Published:** 2025-10-04

S. Zhao, X. Lin, Y. Huang, et al., “Prognostic Value of Ubiquitination-Related Genes in Ovarian Cancer and Their Correlation With Tumor Immunity,” *Human Mutation* 2025 (2025): 8369299, https://doi.org/10.1155/humu/8369299.

In the article titled “Prognostic Value of Ubiquitination-Related Genes in Ovarian Cancer and Their Correlation With Tumor Immunity,” there were errors in all the affiliations. The corrected affiliation list appears below:

Shu Zhao,^1,2,3,4^ Xiaojing Lin,^1,3^ Yuying Huang,^2^ Zhongmin Kang,^5^ Huali Luo,^2^ Qizhu Zhang,^1,3^ Qinshan Li,^1,2,3^ and Mengxing Li^5^

*^1^Department of Obstetrics and Gynecology, Institute of Precision Medicine of Guizhou Province, Affiliated Hospital of Guizhou Medical University, Guiyang, Guizhou, China*

*^2^Department of Clinical Biochemistry, School of Medical Laboratory Science, Guizhou Medical University, Guiyang, Guizhou, China*

*^3^Clinical Medical College, Guizhou Medical University, Guiyang, Guizhou, China*

*^4^Department of Obstetrics, Guizhou Provincial People's Hospital, Guiyang, Guizhou, China*

*^5^Department of Hematology, Guizhou Province Institute of Hematology, Guizhou Province Laboratory of Hematopoietic Stem Cell Transplantation Centre, Affiliated Hospital of Guizhou Medical University Guiyang, Guizhou, China*

We apologize for this error.

