# Supplementary material for: Prognostic Value of Ubiquitination-Related Genes in Ovarian Cancer and Their Correlation With Tumor Immunity
Source: Hum Mutat. 2025 Jul 15;2025:8369299. doi: 10.1155/humu/8369299 (PMC12283211; doi:10.1155/humu/8369299)
Supplement: Supporting Information — Additional supporting information can be found online in the Supporting Information section. Figure S1: Screening and categorization of OS-related UbRGs in OC. (S1A) PCA suggested that the UbRG levels were able to distinctly separate individuals with TCGA-OV into two cohorts: Cohort 1 and Cohort 2. (S1B) GO enrichment analysis was conducted on key prognostic genes. (S1C) KEGG pathways for these key prognostic genes were identified. Figure S2: Experimental flow chart. Table S1: Reagents are mainly used. [file 8369299.f1.docx]

**Supplementary materials**


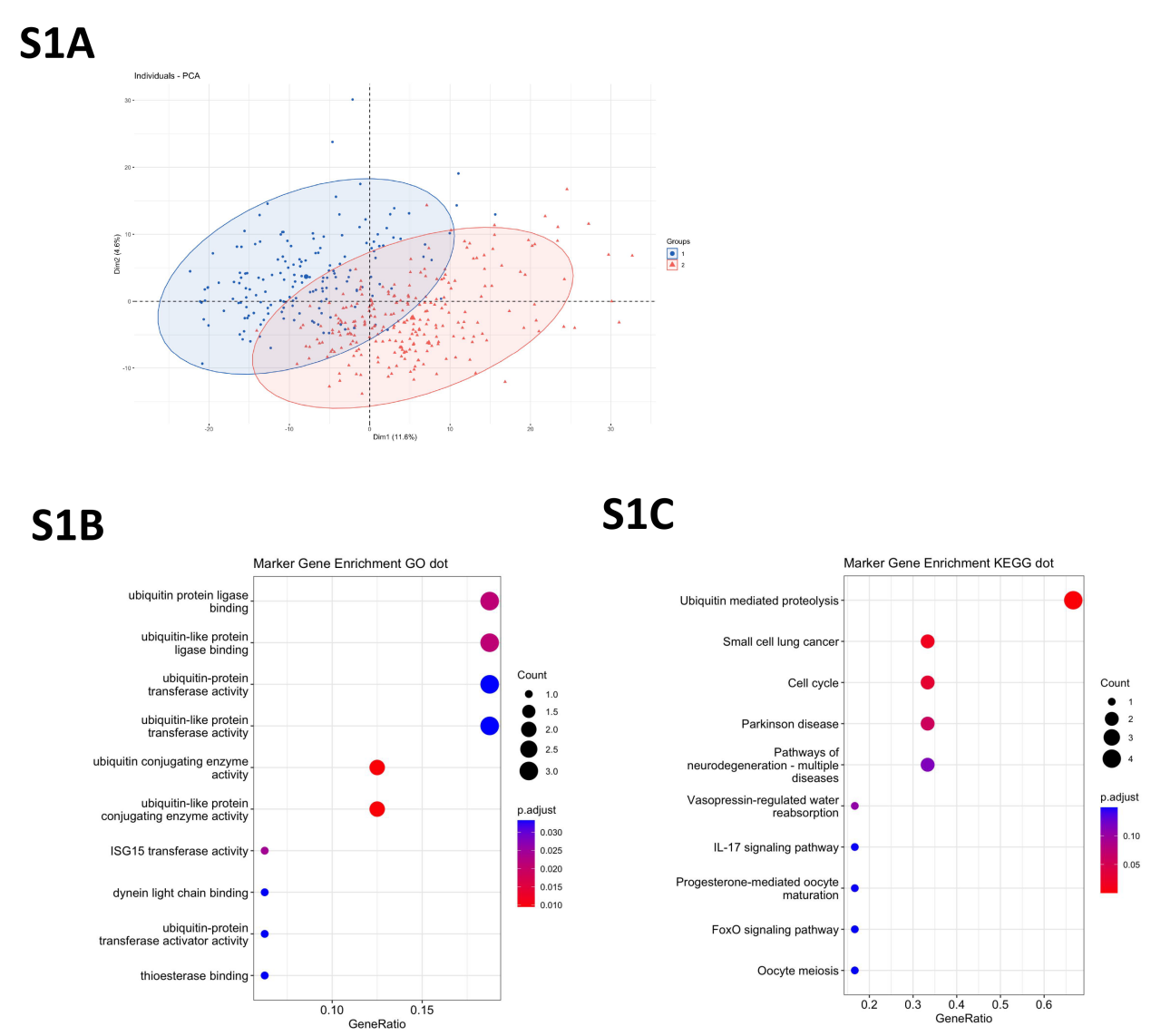


**Supplemental Figure 1.** Screening and categorization of OS-related UbRGs in OC.

(S1A) PCA suggested that the UbRG levels were able to distinctly separate individuals with TCGA-OV into two cohorts: cohort 1 and cohort 2. (S1B) GO enrichment analysis was conducted on key prognostic genes. (S1C) KEGG pathways for these key prognostic genes were identified.


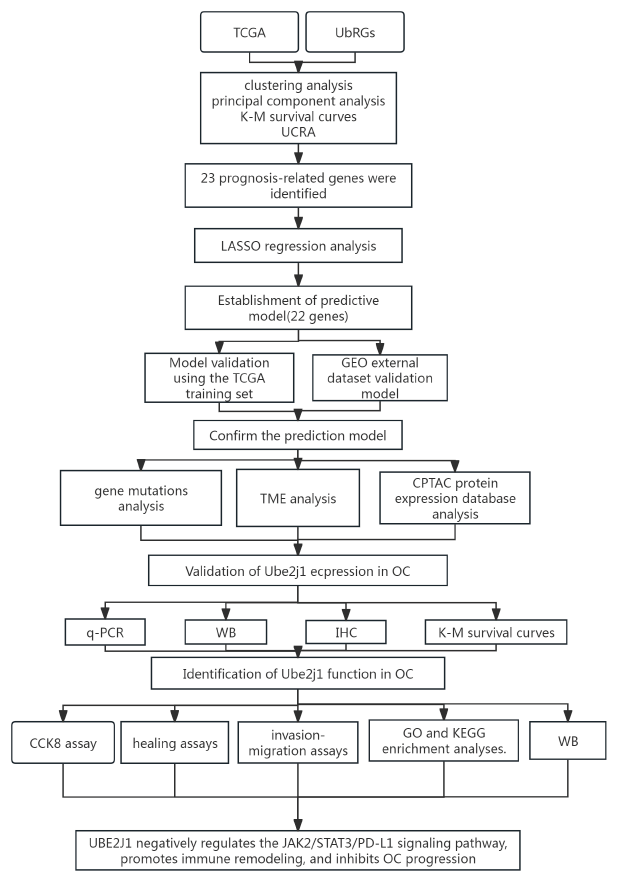


**Supplemental Figure 2.** Experimental flow chart

**Supplemental Table 1.** Reagents are mainly used

| Reagents | Manufacturer/Source |
| --- | --- |
| Primers | Shenggong Bioengineering，Shanghai，China |
| Cells | Basic Medical Sciences Institute’s Cell Center at the  Chinese Academy of Medical Sciences |
| Anti-Smu1 | abclonol, Beijing, China |
| Anti-Ube2j1 | abclonol, Beijing, China |
| Anti-GAPDH | Bioworld, Nanjing, China |
| Cell Counting Kit-8 assay | MCE,South Brunswick Township, NJ, USA |
| 24-well transwells | BD Biosciences, Bedford, MA, USA |
| 12-well plate | BD Biosciences, Bedford, MA, USA |
| PMSF | Biyuntian，Shanghai ，China |
| Hrp-labeled sheep anti-rabbit IgG secondary antibody | Sanying,Wuhan ,China |
| Penicillin/Streptomycin mixture | Gibco,USA |
| Membrane regeneration solution | Soleibao，Beijing ，China |
| 5×protein loading buffer | Solabao,Beijing ,China |
| High-efficiency RIPA Lysis Buffer | Solabao,Beijing ,China |
| Real-time fluorescence quantitative PCR kit | Takara,Japan |
| RNA Reverse Transcription Kit | Takara,Japan |
| Fetal bovine serum | BI， |
| High sugar type DMEM base culture medium | Gibco,USA |
| PVDF | Millipore，USA |
| Lip8000 | Biyuntian，Shanghai，China |
